# An Overview of Pest Species of *Bactrocera* Fruit Flies (*Diptera*: *Tephritidae*) and the Integration of Biopesticides with Other Biological Approaches for Their Management with a Focus on the Pacific Region

**DOI:** 10.3390/insects6020297

**Published:** 2015-04-03

**Authors:** Roger I. Vargas, Jaime C. Piñero, Luc Leblanc

**Affiliations:** 1 Daniel K. Inouye, U.S. Pacific Basin Agricultural Research Center, Agricultural Research Service, United States Department of Agriculture, 64 Nowelo St., Hilo, HI 96720, USA; 2 Cooperative Research and Extension, Lincoln University, 900 Chestnut St., Allen Hall 212, Jefferson City, MO 65101, USA; E-Mail: pineroj@lincolnu.edu; 3 Department of Plant and Environmental Protection Sciences, College of Tropical Agriculture and Human Resources, University of Hawaii, 3050 Maile Way, Room 310, Honolulu, HI 96822, USA; E-Mail: leblancl@ctahr.hawaii.edu

**Keywords:** area wide control, reduced risk insecticides, IPM, *Bactrocera dorsalis*, *Bactrocera cucurbitae*, *Bactrocera tryoni*

## Abstract

Fruit flies (*Diptera*: *Tephritidae*) are among the most economically important pest species in the world, attacking a wide range of fruits and fleshy vegetables throughout tropical and sub-tropical areas. These species are such devastating crop pests that major control and eradication programs have been developed in various parts of the world to combat them. The array of control methods includes insecticide sprays to foliage and soil, bait-sprays, male annihilation techniques, releases of sterilized flies and parasitoids, and cultural controls. During the twenty first century there has been a trend to move away from control with organophosphate insecticides (e.g., malathion, diazinon, and naled) and towards reduced risk insecticide treatments. In this article we present an overview of 73 pest species in the genus *Bactrocera,* examine recent developments of reduced risk technologies for their control and explore Integrated Pest Management (IPM) Programs that integrate multiple components to manage these pests in tropical and sub-tropical areas.

## 1. Introduction

Fruit flies of the family Tephritidae constitute a group of agricultural pests of worldwide importance that attack a wide range of fruits and vegetables [1]. Numerous fruit fly species constitute enormous threats to fruit and vegetable production throughout the world, causing both quantitative and qualitative losses. Furthermore, due to their susceptibility to invasive tephritid species, many fruit-producing countries have imposed quarantine restrictions on the import of products from countries infested with particular fruit fly species, and/or require that fruits and vegetables undergo quarantine treatment before their importation is allowed [2]. Thus, suppression or eradication of fruit flies has often been the goal of control programs.

Integrated Pest Management (IPM) is one method to achieve sustainable agricultural production with less damage to the environment [3]. While IPM has many definitions, it often includes a diverse mix of approaches to manage pests and keep them below economically damaging levels, using control options that range from cultural to chemical components. In practice, IPM ranges from chemically-based systems that involve the targeted and judicious use of synthetic pesticides, to biologically-intensive approaches that manage pests primarily or fully through nonchemical means [4]. In recent years, IPM has been seen as an effective method for managing pestiferous fruit flies in an attempt to make fruit production more sustainable [2].

The genus *Bactrocera* Macquart comprises 651 described species. It is the most economically significant fruit fly genus with at least 50 species considered to be important pests, many of which are highly polyphagous [1,5] (Table 1). The genus *Bactrocera* is widely distributed throughout tropical Asia, the south Pacific and Australia. Relatively few species exist in Africa, and only olive fly, *B. oleae* (Rossi), occurs in Southern Europe [1]. Recently, *B*. *oleae* became established in California and two species in the *B. dorsalis* complex became established on two new continents: *B. carambolae* Drew & Hancock, the carambola fruit fly, in South America (Suriname) and *B. dorsalis* (formerly *B. invadens* Drew, Tsuruta & White) in Africa (Kenya) [6,7]. The oriental fruit fly, *B. dorsalis* (Hendel), is native throughout tropical Asia, and has been recorded from over 270 host plant species [1,5,8].

While IPM of fruit flies has made many unique contributions to agriculture through the incorporation of ecological principles into pest management, truly effective IPM systems are scarce. A literature search performed in ISI Web of Science in early-March, 2015 returned 4841 articles published since 1984 when “Tephritidae” was searched, and 1543 articles were returned when IPM (focusing on agriculture) was used as key word. Surprisingly, the search returned only 54 articles when both “Tephritidae” and “IPM” were searched and less than half of those truly referred to IPM components. By refining these studies by the term “*Bactrocera*”, only 28 articles were returned. Clearly, while different search terms certainly would change the corresponding results, it can be seen that IPM of fruit flies, including *Bactrocera* make up only a small proportion of the overall Tephritidae literature.

Examples of IPM programs targeting *Bactrocera* species include the Regional Fruit Fly Project in the Pacific that targeted *Bactrocera* fruit flies in Pacific Island Countries and Territories [9,10] and the Hawaii Area-Wide IPM program (HAWPM), implemented over a 10-year period in Hawaii. The HAWPM program was not aimed at eradication of fruit flies, but predicated on a pest management strategy that would reduce the entire population in and around cropping areas where economic damage occurred [2]. In practice, implementation of IPM programs targeting fruit flies should be based on a particular crop/pest/environment scenario, IPM goals, e.g., temporal/spatial scales for implementation, knowledge of pest ecology and natural enemies, as well as knowledge of socio-economic factors [11,12]. IPM programs against pestiferous fruit fly species can be implemented at local (e.g., single orchard) and regional (e.g., area-wide IPM) levels and not all IPM components that may be available for a given system would be appropriate or affordable for implementation in small-scale farming operations. In this article we present an overview of the tropical pest species in the genus *Bactrocera,* examine recent developments of reduced risk technologies for control and explore IPM programs that utilize multiple components to manage these pests in tropical and sub-tropical areas.

## 2. Overview of Pest *Bactrocera* Species

**Figure 1:**
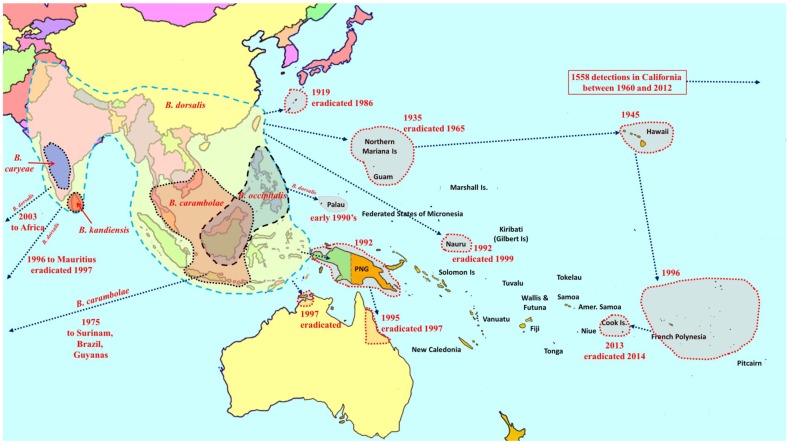
Distribution and invasion history of species in the Oriental fruit fly (*Bactrocera dorsalis*) complex.

**Figure 2:**
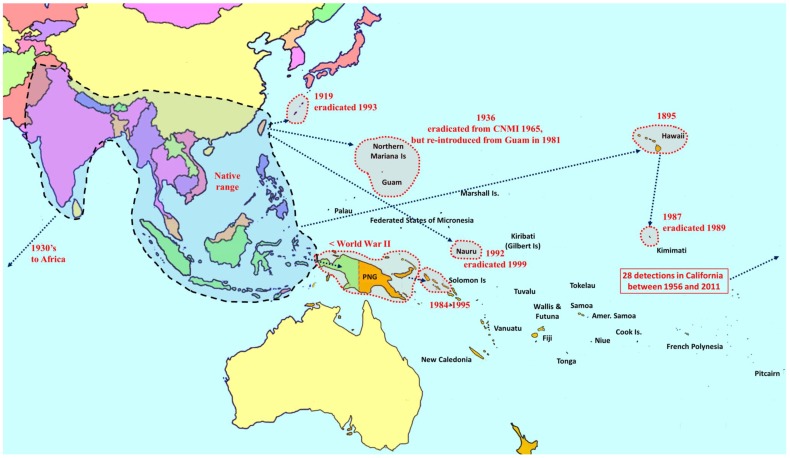
Distribution and invasion history of melon fly (*Bactrocera cucurbitae*).

**Figure 3:**
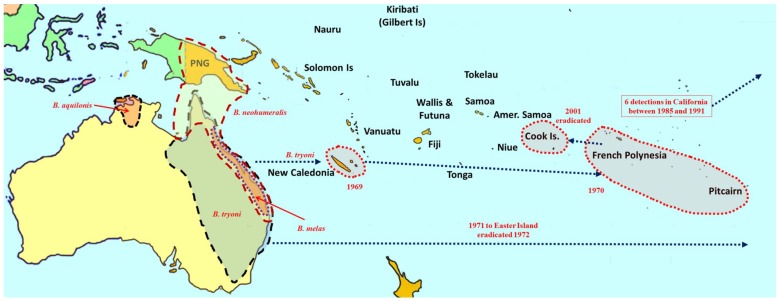
Distribution and invasion history of species in the Queensland fruit fly (*Bactrocera tryoni*) complex.

**Figure 4:**
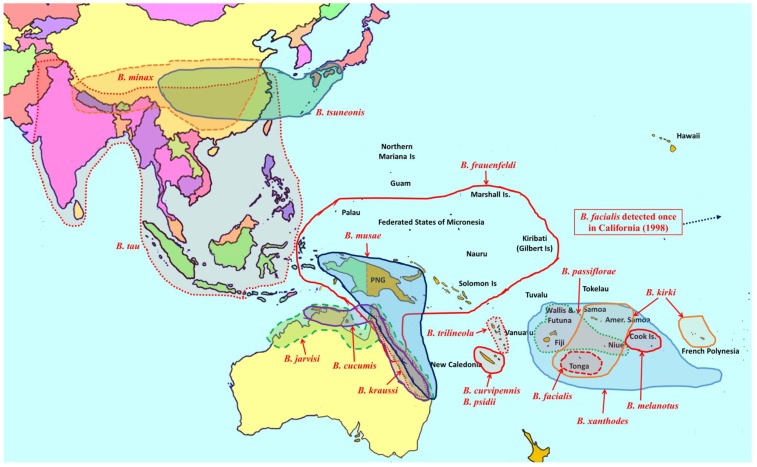
Distribution of Category B pest species in the genus *Bactrocera*.

Seventy-three species of *Bactrocera* have been reared from commercial and/or edible host fruit, hence they are treated here as destructive or potential pests, out of a total of 210 species reared from over 811 host species [8,13,14,15,16,17,18,19]. Individual species are generalist or specialist pests of fruit (57 species). Some species infest fruit and/or flowers of cucurbits (16 species). All 73 *Bactrocera* species that are economically important are listed in Table 1, ranked under four categories, based on pest severity, host range, invasiveness, and frequency of infestation. Category A includes widespread invasive polyphagous generalists or highly destructive specialists that have become established outside of their native range (Figure 1, Figure 2 and Figure 3). Category B pests are polyphagous fruit pests or destructive specialists more restricted in distribution, but at elevated risk of spreading to new locations (Figure 4). Under category C we list relatively minor oligophagous or specialist fruit or cucurbit pests. Category D includes species that have been occasionally bred from commercial/edible fruit or cucurbits.

Among the most destructive Category A species are *B. dorsalis* (oriental fruit fly), *B*. *cucurbitae* (Coquillett) (melon fly) and *B. tryoni* (Froggatt) (Queensland fruit fly). The *B. dorsalis* complex is a large group composed of 85 species [14,16,20], of which five are polyphagous fruit pests (Figure 1). Recently, *B. philippinensis* Drew and Hancock was declared a synonym of *B. papayae* Drew and Hancock [16], and the latter was in turn, along with *B. invadens*, declared a synonym of *B. dorsalis* [21]. This regrouping has greatly increased the geographic range of *B. dorsalis*. Two of the most destructive species in the complex (*B. dorsalis* and *B. carambolae*) have invaded and become established in the Pacific region, Africa, South America, and debatably in California [22,23] (Figure 1). *Bactrocera cucurbitae* is primarily a pest of cucurbits, and females can infest unripe fruit and flowers. It is also a pest of papaya and infrequently infests other non-cucurbit hosts as well. It is native to Southeast Asia, and was introduced into Africa and parts of Oceania (Figure 2). The *B. tryoni* complex is composed of four very closely related species. While Queensland fruit fly (*B. tryoni*) and the lesser Queensland fruit fly (*B. neohumeralis* (Hardy)), both sympatric, are genetically indistinguishable, yet reproductively isolated by time of mating (dusk for the former and day time for the latter), *B. aquilonis* (May) and *B. melas* (Perkins & May) may be conspecific variants of *B. tryoni* [24]. In the early 1970s, *B*. *tryoni* was introduced and became established in New Caledonia and French Polynesia (Figure 3).

**Table 1:** List of pest species of *Bactrocera*, ranked by category of severity.

Species	Hosts	Distribution
**CATEGORY A pests**		
*B. carambolae* Drew & Hancock	Polyphagous fruit pest	Vietnam to Indonesia. Introduced into South America.
*B. correcta* (Bezzi)	Polyphagous fruit pest	Pakistan to Vietnam.
*B. cucurbitae* (Coquillett)	Primarily Cucurbitaceae (fruit & flower)	Tropical Asia (widespread). Introduced into Africa and Oceania.
*B. dorsalis* (Hendel)	Polyphagous fruit pest	Tropical Asia (widespread). Introduced into Africa and Oceania.
*B. latifrons* (Hendel)	Mainly Solanaceae	Pakistan to Taiwan; south to Sulawesi. Introduced into Hawaii and Africa.
*B. neohumeralis* (Hardy)	Polyphagous fruit pest	Australia, New Guinea.
*B. oleae* (Gmelin)	Olive	Africa. Introduced into southern Europe, the Middle East and California.
*B. tryoni* (Froggatt)	Polyphagous fruit pest	Australia. Introduced in Oceania.
*B. zonata* (Saunders)	Polyphagous fruit pest	India to Vietnam.
**CATEGORY B pests**		
*B. aquilonis* (May)	Polyphagous fruit pest	Australia. May be conspecific with *B. tryoni*.
*B. caryeae* (Kapoor)	Oligophagous fruit pest	Southern India.
*B. cucumis* (French)	Cucurbitaceae (fruit)	Australia.
*B. curvipennis* (Froggatt)	Polyphagous fruit pest	New Caledonia.
*B. facialis* (Coquillett)	Polyphagous fruit pest	Tonga.
*B. frauenfeldi* (Schiner)	Polyphagous fruit pest	Australia, Micronesia (except Marianas), New Guinea, Solomon Islands.
*B. jarvisi* (Tryon)	Polyphagous fruit pest	Australia.
*B. kandiensis* Drew & Hancock	Oligophagous fruit pest	Sri Lanka.
*B. kirki* (Froggatt)	Polyphagous fruit pest	French Polynesia, Fiji (Rotuma), Niue, Samoa (American & Western), Tonga.
*B. kraussi* (Hardy)	Polyphagous fruit pest	Australia.
*B. melanotus* (Coquillett)	Polyphagous fruit pest	Cook Islands.
*B. minax* (Enderlein)	Citrus	Bhutan, China, Nepal.
*B. musae* (Tryon)	Banana	Australia, New Guinea.
*B. occipitalis* (Bezzi)	Oligophagous fruit pest	Kalimantan, Philippines.
*B. passiflorae* (Froggatt)	Polyphagous fruit pest	Fiji, Wallis & Futuna, Niue.
*B. psidii* (Froggatt)	Polyphagous fruit pest	New Caledonia.
*B. tau* (Walker)	Cucurbitaceae (fruit)	Pakistan to Philippines; south to Sumatra & Sulawesi.
*B. trilineola* Drew	Polyphagous fruit pest	Vanuatu.
*B. tsuneonis* (Miyake)	Citrus	China, Japan.
*B. xanthodes* (Broun)	Polyphagous fruit pest	Cook Islands, Fiji, French Polynesia (Austral group), Niue, Samoa (American & Western), Tonga, Wallis & Futuna.
**CATEGORY C pests**		
*B. albistrigata* (deMeijere)	Oligophagous fruit pest	Indonesia, Malaysia
*B. atrisetosa* (Perkins)	Cucurbitaceae (fruit)	New Guinea.
*B. bryoniae* (Tryon)	Banana, chili pepper	Australia, New Guinea.
*B. caudata* (Fabricius)	Cucurbitaceae (flowers)	India to Taiwan; south to Indonesia (Lesser Sundas).
*B. decipiens* (Drew)	Cucurbitaceae (fruit)	New Guinea.
*B. depressa* (Shiraki)	Cucurbitaceae (fruit)	Japan, South Korea, Taiwan.
*B. distincta* (Malloch)	Sapotaceae	Fiji, Samoa (American & Western), Tonga, Wallis Is.
*B. diversa* (Coquillett)	Cucurbitaceae (flowers)	Pakistan to Vietnam.
*B. halfordiae* (Tryon)	Oligophagous fruit pest	Australia.
*B. melas* (Perkins & May)	Polyphagous fruit pest	Australia. May be conspecific with *B. tryoni*.
*B. moluccensis* (Perkins)	*Inocarpus fagifer*	Java to New Guinea, Solomon Islands.
*B. obliqua* (Malloch)	Guava, *Syzygium*	New Guinea.
*B. passiflorae* (sp. nr.)	Oligophagous fruit pest	Fiji, Tokelau, Tonga (Niuas Group), Tuvalu.
*B. pyrifoliae* Drew & Hancock	Guava, peach, pear	Thailand, Vietnam. (Member of *B. dorsalis* complex).
*B. scutellaris* (Bezzi)	Cucurbitaceae (flowers)	India to Vietnam; south to peninsular Malaysia.
*B. scutellata* (Hendel)	Cucurbitaceae (flowers)	Bhutan to Vietnam; north to Taiwan & Japan (Ryukus).
*B. strigifinis* (Walker)	Cucurbitaceae (flowers)	Australia, New Guinea.
*B. triangularis* (Drew)	Cucurbitaceae (flowers)	New Guinea.
*B. trivialis* (Drew)	Oligophagous fruit pest	New Guinea. (Member of *B. dorsalis* complex).
*B. tuberculata* (Bezzi)	Oligophagous fruit pest	Bangladesh to Vietnam.
*B. umbrosa* (Fabricius)	Breadfruit, jackfruit	Widespread from southern Thailand through New Guinea to New Caledonia.
**CATEGORY D pests**		
*B. arecae* (Fabricius)	Betel nut	Malaysia (Peninsular), Singapore, Thailand.
*B. atramentata* (Hering)	*Pometia pinnata*	New Guinea.
*B. bancroftii* (Tryon)	Mulberry	Australia.
*B. expandens* (Walker)	Mangosteen	Australia, Indonesia (Moluccas), New Guinea.
*B. hastigerina* (Hardy)	*Spondias*	New Guinea, Solomon Islands.
*B. hochii* (Zia)	*Luffa cylindrica* (fruit)	Bangladesh to Vietnam; south to Sumatra.
*B. lineata* (Perkins)	*Pometia pinnata*	New Guinea.
*B. mesomelas* (Bezzi)	Guava	Africa.
*B. mucronis* (Drew)	Guava, sweetsop	New Caledonia.
*B. munda* (Bezzi)	Squash (fruit)	Philippines, Taiwan.
*B. murrayi* (Perkins)	Mango, Surinam cherry	Australia, New Guinea.
*B. mutabilis* (May)	Guava, kumquat	Australia.
*B. nigrofemoralis* White & Tsuruta	Pomelo, mamey sapote	Indian subcontinent, including Sri Lanka.
*B. nigrotibialis* (Perkins)	Guava, rose-apple	India to Vietnam; south to Indonesia (Lesser Sundas).
*B. ochroma* Drew & Romig	Mango	Indonesia.
*B. perfusca* (Aubertin)	Mango, rose-apple	French Polynesia (Marquesas only).
*B. pruniae* Drew & Romig	Peach	Vietnam.
*B. quadrisetosa* (Bezzi)	*Pometia pinnata*	Solomon Islands, Vanuatu.
*B. speculifera* (Walker)	Breadfruit	New Guinea.
*B. tapervitta* Mahmood	*Luffa cylindrica* (fruit)	Philippines.
*B. trichosanthes* Drew & Romig	Bittergourd (fruit)	Indonesia (Java), Malaysia (Peninsular & East), Thailand.
*B. trimaculata* (Hardy & Adachi)	Bittergourd (fruit)	Philippines.
*B. versicolor* (Bezzi)	Sapodilla	India, Sri Lanka.

## 3. Area-Wide Control Technologies

Various technologies have been developed for area-wide control of *Bactrocera* fruit flies and related species throughout Asia and the Pacific. These include: (1) Insecticide-based suppression tools including cover sprays [25], protein bait sprays [2,26,27,28,29] and soil drenches [30,31,32]; (2) Male annihilation [33,34,35,36]; (3) Sterile insect releases [37,38,39]; (4) Releases of natural enemies [40,41]; and (5) cultural controls [9,42,43]. When used alone, however, these tactics do not constitute, by definition, Integrated Pest Management.

### 3.1. Insecticide Cover Sprays, Protein Bait Sprays, and Soil Drenches

The history of fruit fly control with full cover sprays started with inorganic insecticides (e.g., lead arsenate) in the early 1900s and spanned the century with a transition to synthetic insecticides, such as chlorinated hydrocarbons, organophosphates, and synthetic pyrethroids. Advantages of insecticide cover sprays are that they are affordable, convenient and provide a high level of protection against fruit fly infestation with consistent results [42].

Addition of protein food baits to insecticide sprays reduced the amount of pesticide needed for fruit fly control and has been used successfully in many eradication programs [25,26,44]. Female flies, in need of protein for full ovarian development and egg production, readily feed on a protein source containing a toxicant. Enzymatic protein hydrolysate baits were first used in Hawaii for control of *B. dorsalis*, and malathion became the organophosphate insecticide additive of choice due to its low mammalian toxicity, affordable price, and low levels of fruit fly resistance [25,45]. In the 1960s, one particular formulation for ground, aerial and quarantine applications was adopted and remains a common standard today for control of many fruit fly species. It consists of three to four parts Nu-Lure^®^ Insect Bait (Miller Chemical and Fertilizer Corporation, Hanover, PA, USA) plus one part malathion [26]. Because organophosphate insecticides have been implicated in negative effects on natural enemies and human health, alternatives have been sought during the twenty first century. The Regional Fruit Fly Project in the Pacific introduced the more rain-fast fipronil-based Bactrogel formulation, mixed with a protein as a bait spray for use against *Bactrocera* fruit flies in Pacific Island Countries and Territories [9,10]. In the late 1990s, a new bait spray formulation containing the reduced-risk biopesticide spinosad was shown to be as effective as Nulure-malathion bait sprays for use in Central America and the USA against *Ceratitis capitata* (Wiedemann) [27,46]. According to the United States Environmental Protection Agency (EPA), biopesticides are “*certain types* *of pesticides derived from such natural materials as animals, plants, bacteria, and certain minerals*”. Research is also underway to incorporate plant essential oils into protein baits [47].

Spinosad, a toxin derived from a soil-dwelling actinomycete bacterium (*Saccharopolyspora spinosa* Mertz & Yao), has low mammalian toxicity and reduced environmental impact on natural enemies [48]. A spinosad-based hydrolysed protein bait that attracted, induced feeding and killed fruit flies was initially developed by Moreno and Mangan [49]. Later it became known as GF-120 Fruit Fly Bait (Dow AgroSciences, Indianapolis, IN) [40,50]. Traditionally, *B*. *cucurbitae* has been controlled in agricultural areas of Hawaii by using protein bait sprays on border crops [51]. GF-120 was tested and shown to be an adequate replacement for organophosphates through the Hawaii Area-Wide Fruit Fly program for *B*. *cucurbitae* [28], *B*. *dorsalis* [52] and *B*. *latifrons* (Hendel).

Another component in many tephritid fly area-wide IPM programs has been the application of insecticidal soil drenches under host trees where fruit flies have been detected [53] and as a regulatory treatment in the certification process for movement of nursery stock outside of fruit fly quarantine areas [31]. Presently, to ship fruit tree nursery stock out of California during fruit fly quarantines, container pots with fruit seedlings and small trees must be drenched with diazinon prior to shipment. In addition, Florida uses diazinon as a soil drench underneath host plants where fruit fly larvae or gravid adult females are detected during eradication programs. Although many uses of diazinon have been discontinued in the United States due to problems with its effects on aquatic organisms in freshwater ecosystems, it is still used in California through a Special Local Needs (SLN) registration and in Florida through an Emergency Crisis Exemption (Section 18). Diazinon is an organophosphate insecticide now banned for outdoor residential use by the EPA [31]. It is not known how much longer the present SLN or Section 18 will continue to be approved in California and Florida. In response to the situation, Stark *et al.* [31,32] identified several efficacious insecticides to replace diazinon as a soil treatment for orchard and back yard fruit tree drenches in nursery containers against fruit flies. Three synthetic pyrethroid insecticides were the most effective products, but the results with a newly formulated spinosad product, Entrust SC™, were also particularly noteworthy. This newly formulated product outperformed the conventional Entrust, and can be used in organic agriculture. Another biopesticide with activity against larvae and pupae of major *Bactrocera* (*B*. *dorsalis* and *B*. *cucurbitae*) and *Ceratitis* (*C*. *capitata*, *C*. *cosyra* (Walker)) species in soil is the fungal pathogen *Metarhizium anisopliae* (Metchnikoff) Sorokin [53].

### 3.2. Male Annihilation

At least 324 species of the male *Bactrocera* are attracted to cue-lure (C-L) (4-(p-acetoxyphenyl)-2-butanone)/raspberry ketone (RK) (4-(p-hydroxyphenyl)-2-butanone) and 123 species to methyl eugenol (ME) (4-allyl-1, 2-dimethoxybenzene-carboxylate) [5,36]. Of the 73 pest *Bactrocera* listed in Table 1, 34 respond to C-L/RK and 16 to ME [5]. Recently, Vargas *et al.* [36] summarized the history of Male Annihilation Technique (MAT) and the many successful programs worldwide. In all cases, eradication was attempted or achieved through repeated area-wide applications, by airdrops and/or manual ground applications, of bait stations (fiberboard or coconut husk blocks, cotton string or wick, or molded paper pulp) impregnated with male lures mixed with a toxicant (naled, malathion, or fipronil). Briefly, following the successful initial suppression experiments in Hawaii [54], the first fruit fly eradication using ME-MAT was achieved with *B. dorsalis* in the Mariana Islands [33]. ME-MAT alone was initially used on Rota; however, sterile insect releases, successful alone at eradicating *B*. *dorsalis* from Guam, had to be augmented with ME-MAT to achieve eradication on the other islands after one year of sterile fly releases [37]. In a larger-scale program, *B*. *dorsalis* was eradicated from the Ryuku Islands [34]. Although not as powerful as ME as an attractant, C-L-MAT was used with some degree of success, such as in the eradication of *B. tryoni* from Rapa Nui (Easter Island) in the southern Pacific using a combined treatment of C-L and malathion on pieces of cotton string and spot spraying with protein–malathion bait spray in the 1970s [55].

From 1960 to 2012, nine different *Bactrocera* species have been detected in California, with *B*. *dorsalis* detected most frequently (126 times). Altogether, there have been 140 eradication programs with 25 quarantines [36]. Florida has had 12 *B*. *dorsalis* detections since 1999 (in seven separate years) with the last occurring in 2010 [36]. In response to these constant introductions, reduced-risk MAT treatments have been developed. In initial small-scale trials in Hawaii and subsequent weathering trials in California, Vargas *et al.* [56,57] provided evidence that reduced-risk formulations of SPLAT with ME and spinosad (AKA SPLAT-MAT-spinosad-ME (ISCA Technologies, Riverside, CA USA) or STATIC^TM^ (Dow AgroSciences, Inianapolis, IN USA) Spinosad-ME) performed as well as or better than standard Min-U-Gel with naled [58]. STATIC^TM^ Spinosad-ME has been tested and registered for use in California and Florida and offers a novel and convenient ready-to-use MAT formulation that is safer than the current use of organophosphates that may pose potential negative effects on human and environmental health [36,59,60]. A SPLAT-MAT-C-L formulation has also been tested successfully in Hawaii [56] for use against C-L responding flies.

### 3.3. Sterile Insect Releases

Releases of sterile males for suppressing wild populations of the same species was first proposed by E.F. Knipling [61], and effectively implemented against *Cochliomyia hominivorax* (Coquerel), the New World screwworm fly [62]. This technique became known as the sterile insect technique (SIT) [63], and is well suited for the suppression or eradication of *Bactrocera* flies that display elaborate courtship behavior that can be mediated by male lures [64,65]. Scientists from Hawaii and Australia carried out the original pilot SIT tests to eradicate *B*. *cucurbitae* from Rota in the Northern Mariana Islands [66], suppress *B. tryoni* in Australia [67], eradicate *B*. *dorsalis* in Micronesia [37] and suppress or eradicate *C*. *capitata* in Hawaii [68] and California [69]. SIT has since become the method of choice for eradication of *C. capitata* outbreaks in California and Florida. In Japan, *B*. *cucurbitae* was eradicated by SIT [38] using the approach developed in Hawaii. A major improvement and cost saving came with the development of laboratory strains (e.g., *C*. *capitata*) that allowed for rearing “males-only” flies for SIT programs. The advantages of releasing solely males included avoidance of ‘sting-damage’ by sterile females and avoidance of mating between sterile males and sterile females. During the HAWPM program, development of a sexing strain for *B*. *cucurbitae* [70] allowed for the application of SIT to small-farm situations in selected areas and reduced the local *B. cucurbitae* population to near extinction [39]. Although very successful in demonstration trials, the need for a large mass-rearing facility in Hawaii and more cost-effective ‘sexing strains,’ limited its implementation. Nonetheless, the “males-only” technology allowed for use of sterile flies as part of IPM programs against *Bactrocera* species.

### 3.4. Releases of Natural Enemies

The role of parasitoids in the HAWPM program was examined at three levels of application: (i) conservation; (ii) classical releases; and (iii) augmentative releases [2,71]. An overall goal of the program was to conserve biological control in economic crops through the use of reduced-risk insecticides such as GF-120 Fruit Fly Bait and male annihilation using bucket traps in an IPM approach [2,52]. The program succeeded in both reducing the use of organophosphates and conserving biological controls, such as *Fopius arisanus* (Sonan) and related braconid species, while suppressing fruit flies below economic injury levels. Hawaii has a long history of the application of biological control against introduced insect pests. This is also true for tephritid flies where classical biological control started soon after the first flies were established in Hawaii [72]. Recently, Vargas *et al.* [41] reviewed the major contributions of various scientists in Hawaii to biological control of fruit flies worldwide.

### 3.5. Cultural Controls

Cultural controls involve a variety of approaches ranging from use of fruit fly resistant varieties, to early harvesting of fruit, bagging of fruit and field sanitation [9,42,73]. In the case of tephritid fruit flies, cultural practices can support synergies with other components of IPM, such as protein bait sprays and biological control. Here, we discuss the types of cultural control methods that have been evaluated/implemented, either alone or in combination with other IPM tools, to suppress populations of *Bactrocera* species.

#### 3.5.1. Sanitation

Field sanitation is a technique that either prevents fruit fly larvae from developing or sequesters young emerging adult flies so that they cannot return to the crop to reproduce [2]. There are various ways of achieving this end. One is the removal and disposal of infested or uninfested (cull) produce. While this can be laborious, it is a very effective fruit fly suppression method [74,75] and a key component of an IPM program for fruit flies. The importance of field sanitation was recently demonstrated by Piñero *et al.* [29]. These authors developed quantitative (by counting numbers of ground fruit) and qualitative (by developing a ranking system) approaches to measure level of implementation of field sanitation in papaya orchards in Hawaii and the impact of field sanitation on fruit fly population densities and fruit infestation. In this large-scale study, numbers of female *B. dorsalis* captured in monitoring traps and also levels of fruit infestation were correlated with variations in sanitation levels in the experimental plots. For instance, significantly more female *B. dorsalis* were captured in experimental plots that were categorized as having poor sanitation than either in good sanitation plots or in forested areas. As sanitation improved in subsequent weeks, numbers of females/trap/day in poor sanitation plots dropped substantially, though they were still significantly higher than good sanitation plots.

#### 3.5.2. Fruit Bagging

The simple action of wrapping individual fruits has proven effective at preventing fruit infestation by fruit flies. Wrapping materials can be newspaper, paper bags, or polythene sleeves in the case of long/thin fruits [76]. This system also provides physical protection from mechanical injuries (scars and scratches) and, in some cases, reduces fungal spots on the fruits. Although laborious, it is cheaper, safer, easier to do, and gives farmers a more reliable estimate of projected harvest.

#### 3.5.3. Augmentorium

The so-called augmentorium is a tent-like structure developed by USDA ARS researchers in Hawaii with the purpose of enclosing fruits and/or vegetables infested with fruit fly larvae. By having a fine mesh, the structure keeps emerging fruit flies inside but allows beneficial parasitoids to escape [43]. Augmentoria have proven effective at reducing fruit fly populations when compared with trapping [43]. Further details on this method of sanitation used in the HAWPM fruit fly program are found in Klungness *et al.* [43,77]. More recent research with augmentoria has been conducted in Reunion Island. In a study conducted by Deguine *et al.* [78], augmentoria with a mesh having a hole area of 3 mm^2^ prevented 100% of adult *B. cucurbitae* from escaping while 100% of the parasitoids (*Psyttalia fletcheri* (Silvestri) and *Fopius arisanus*) were able to escape from the mesh. Thus, this method is an excellent way of removing pestiferous fruit flies while conserving natural enemies.

#### 3.5.4. Soil Disturbance

Plowing and ground flooding are two ways in which fruit fly pupae in the soil can be exposed to environmental conditions leading to increased mortality. Few reports exist in the literature on the effects of soil disturbance on suppression of *Bactrocera* species. In one study, Verghese *et al.* [79] reported that three-weekly inter-tree plowing and raking was used as part of an IPM package that included field sanitation and cover sprays of insecticide. The effectiveness of this package as implemented by mango producers in India was recorded over a nine-year period. Infestation reductions attributable to the IPM package were between 77% and 100% in different years. However, efficacy was evaluated for the entire IPM package, therefore the effectiveness of each of the IPM components is unclear.

## 4. Examples of Successful Fruit Fly IPM Systems against *Bactrocera* spp.

Fruit fly IPM systems range from programs for individual homeowners and farmers to large areas of many square kilometers. During the twenty first century the Regional Fruit Fly Project in the Pacific pioneered the implementation of sustainable technologies throughout many Pacific Island Countries for control of *Bactrocera* fruit flies [9,10]. These technologies included fipronil-based bait sprays and male annihilation treatments, in conjunction with cultural controls. Similarly, the HAWPM program tested and demonstrated larger IPM programs to control *B. dorsalis* and *B. cucurbitae* that included: (1) field sanitation, (2) protein bait, (3) lures, (4) SIT, and (5) biological control [2]. This program registered many technologies for farmers and homeowners and promoted the use of safer or reduced risk fruit fly protein baits and MAT traps in what became popularly referred to as the “1 (sanitation), 2 (protein bait), 3 (male lure trapping) approach” for fruit fly control [80]. For example, in a study that aimed at assessing the efficacy of GF-120 NF Naturalyte Fruit Fly Bait sprays in conjunction with field sanitation to control *B. dorsalis* in papaya orchards in Hawaii, Piñero *et al.* [52] reported significant reductions in numbers of female *B. dorsalis* captured by monitoring traps and in levels of infestation of papaya fruit by *B. dorsalis* only when both GF-120 was applied in a sustained manner in conjunction with field sanitation and male annihilation.

Asian and African countries have also demonstrated the ability to control major pest species, with some examples from India presented here. As discussed above, Verghese *et al.* [79,81] evaluated the effectiveness of an IPM package targeting *B. dorsalis* in mango orchards in India with good results, integrating MAT, field sanitation and insecticide sprays. Best results were obtained when MAT, sanitation and delta-methrin were combined with azadirachtin over a two-year period. In a study conducted in mango orchards in India, Singh *et al.* [82] reported that *B. dorsalis* and *B. zonata* (Saunders) were effectively suppressed by integrating multiple approaches. Maximum fruit protection (94.5%) was recorded with integration of MAT + sanitation + soil drenching with 0.1% chorpyriphos + bait cover spray (0.05% malathion + 0.2% Protinex). This was followed by a combination of MAT + sanitation + soil drenching (87.3% protection), MAT+ sanitation + cover spray (81.8% protection), and MAT + sanitation (65.5% protection). Clearly, the removal of soil drenching or bait cover sprays reduced the effectiveness of the crop protection program, highlighting the need to include chemical controls into suppression programs of aggressive species, such as *B. dorsalis*. Gogi *et al.* [83] reported a reduction in infestation of *Momordica charantia* L. by *B. cucurbitae*, leading to increased marketable yields through the integration of three components of cultural management: (1) Early sowing, (2) Hand Sowing Method (HSM), and (3) sanitation.

Although *B*. *oleae* is more of a sub-tropical than a tropical pest species, methods to manage it have been similar to tropical species. Integrated control of *B*. *oleae* was proposed soon after the pest was found in the olive production areas of California [84]. Recommendations for commercial orchards included releases of biological control parasitoids (*Psyttalia humilis* (Silvestri), *P*. *concolor* (Szépligeti), and *P. lounsburyi* (Silvestri)), cultural controls, attract-and-kill traps and GF-120 NF Naturalyte Fruit Fly Bait. Sanitation has been a major consideration, accomplished by removing all unharvested fruits and standing water in orchards that provide adults with water [85]. Attract-and-kill traps (Magnet OLI, Suterra, Bend, OR) [86] used as bait stations have shown promise for *B*. *oleae* control and greatly reduce the amount of bait spray applied in olive orchards because they attract the pest to an attractive device that contains the toxicant [87,88].

Most challenging have been the accidental introductions of fruit flies into the US mainland. Recently STATIC^TM^ Spinosad-ME, developed late in the HAWPM program, has been registered in California and Florida for use with GF-120 against accidental introductions of *Bactrocera*. In addition, lambda-cyhalothrin has been tentatively approved as a replacement for diazinon for use as a soil drench in Florida. Research continues on the possible use of Entrust SG as a biopesticidal soil drench as part of a three-pronged area-wide IPM system for control of fruit flies accidentally introduced into the U.S. mainland [32]. One of the largest multi component programs used on the U.S. mainland covers over 5000 km^2^ in California and Florida. The primary technology used is the release of millions of sterile *C*. *capitata* flies. When infestations are found, the program is supplemented with fruit stripping and treatment of host trees with GF-120. This same SIT approach could also be used for suppression of many *Bactrocera* fruit flies (e.g., *B*. *dorsalis* and *B*. *cucurbitae*) if it was not for the effectiveness of MAT.

## 5. Conclusions

During the last 100 years, insecticides for fruit fly control have included inorganic, synthetic and reduced-risk compounds [89]. Insecticides, particularly bait sprays, will continue to be a major component of fruit fly control systems. However, due to political, social, and environmental issues, reduced-risk compounds and biopesticides are being considered as replacements for the organophosphate, carbamate and synthetic pyrethroids currently being used [89]. For example, during the past 15 years, replacement insecticides have been discovered and registered for the organophosphate insecticides malathion (in bait sprays), naled (in MAT treatments), and diazinon (for soil drenches). These products, although often more expensive than their organophosphate counterparts, lend themselves more readily to development of *Bactrocera* area-wide IPM programs that include bait sprays, attract and kill lures (*i.e.*, MAT) as well as cultural and biological components. Although true IPM programs are currently scarce for pest tephritid flies, these new biopesticide and reduced risk compounds may allow for development and expansion of new IPM systems without many of the side effects of conventional synthetic insecticides on the environment. In addition many of these novel biopesticide compounds can be used in insect management systems for production of organic fruits and vegetables.
